# P-1867. Electronic Delivery of an OPAT Patient Education Video: Enhancing Accessibility and Clinician Satisfaction

**DOI:** 10.1093/ofid/ofaf695.2036

**Published:** 2026-01-11

**Authors:** Amy Van Abel, Abinash Virk, Marla R Frahm, Rachel Schmidt, Margaret Pertzborn, Jennifer Lamb, Allyson M Dodson, Christina G Rivera (O'Connor)

**Affiliations:** Mayo Clinic, Lino Lakes, MN; Mayo Clinic, Lino Lakes, MN; Mayo Clinic, Lino Lakes, MN; Mayo Clinic, Lino Lakes, MN; Mayo Clinic Health System, Eau Claire, Wisconsin; Mayo Clinic, Lino Lakes, MN; Mayo Clinic, Lino Lakes, MN; Mayo Clinic, Lino Lakes, MN

## Abstract

**Background:**

Outpatient parenteral antimicrobial therapy (OPAT) has been shown to have numerous benefits including lower costs, increased patient satisfaction, and decreased healthcare associated infections. Patient education is a crucial part of the OPAT process, as lack of understanding may lead to adverse drug events, emergency department visits, or hospital re-admission. A baseline survey of OPAT clinicians at a large multi-state health system revealed low awareness of existing OPAT education resources and consensus that the materials needed improvement. We hypothesized that an OPAT education video would improve OPAT staff satisfaction with the educational offerings.

**Figure 1.**
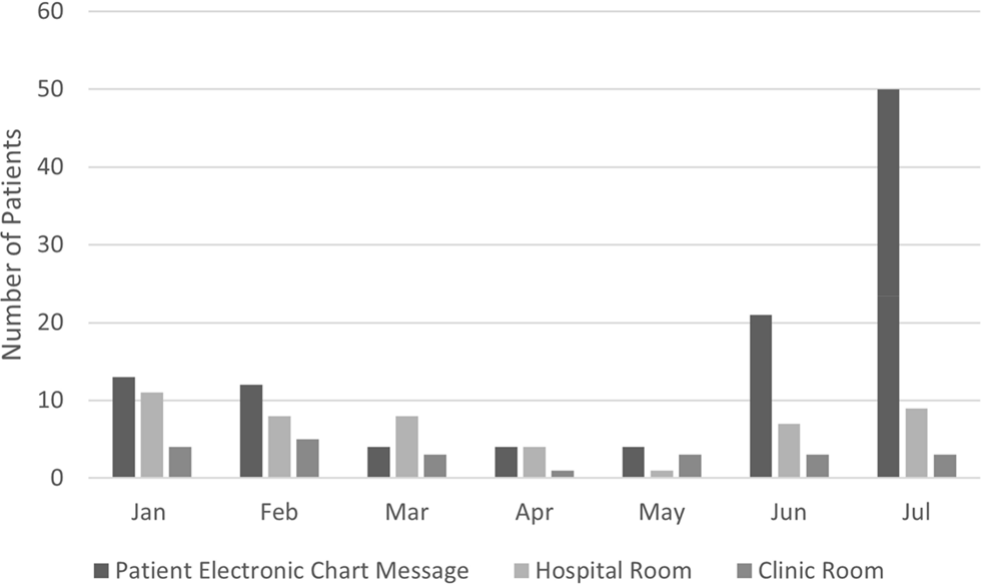
OPAT Video Link Engagement (2024)

**Figure 2.**
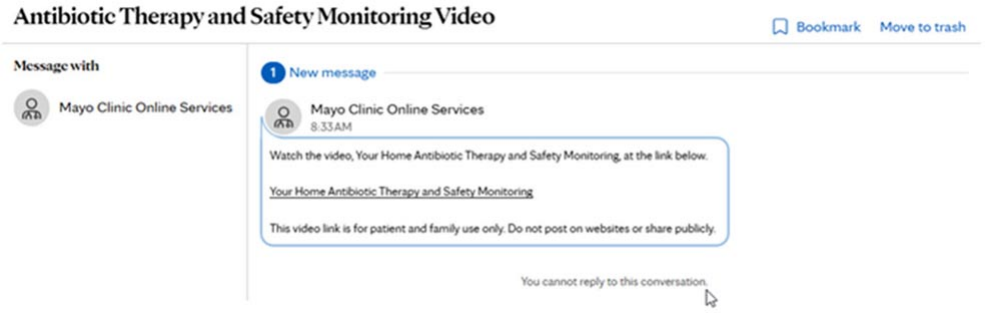
Patient Electronic Chart Message

**Methods:**

Adult patients ( > 18 years of age) enrolled in OPAT programs at 4 sites within Mayo Clinic throughout Minnesota and Wisconsin from 5/1/23 - 8/1/24 were included. A standardized OPAT patient education video was developed using voice over PowerPoint slides. Video content included lab monitoring requirements, signs and symptoms of adverse effects including alarm symptoms, and intravenous catheter care. The video was available as a link for delivery via patient electronic chart message, to be played on clinic and hospital room computers, and accessed by patients via QR code on rack cards. We implemented inclusion rules built within our electronic health record which automated the delivery of these patient chart messages. The primary outcome was staff satisfaction with OPAT education materials. Secondary metrics included number of video patient accesses in hospital and clinic rooms, number of message deliveries, and frequency of message opening. Staff satisfaction with OPAT education was compared pre- and post-intervention using the Pearson chi-square test.

**Results:**

The patient education video was accessed 73 times in hospital and clinic rooms and was delivered by automated patient chart message 157 times during the study period. Patient chart messages were opened 91.5% of the time. The percentage of staff who felt OPAT education materials needed improvement decreased from 90% pre-intervention to 38.1% post-intervention (p=0.05).

**Conclusion:**

A standardized OPAT patient education video allowed for efficient electronic delivery of educational information to patients and improved OPAT staff satisfaction.

**Disclosures:**

All Authors: No reported disclosures

